# An Efficient New Route to Dihydropyranobenzimidazole Inhibitors of HCV Replication

**DOI:** 10.3390/molecules16010281

**Published:** 2010-12-30

**Authors:** Matthew A. Parker, Emily Satkiewicz, Thomas Hermann, B. Mikael Bergdahl

**Affiliations:** 1 Department of Chemistry and Biochemistry, San Diego State University, San Diego, CA 92182, USA; 2 Department of Chemistry and Biochemistry, University of California, La Jolla, San Diego, CA 92093, USA

**Keywords:** HCV, benzimidazole, RNA, replication, inhibitor

## Abstract

A class of dihydropyranobenzimidazole inhibitors was recently discovered that acts against the hepatitis C virus (HCV) in a new way, binding to the IRES-IIa subdomain of the highly conserved 5' untranslated region of the viral RNA and thus preventing the ribosome from initiating translation. However, the reported synthesis of these compounds is lengthy and low-yielding, the intermediates are troublesome to purify, and the route is poorly structured for the creation of libraries. We report a streamlined route to this class of inhibitors in which yields are far higher and most intermediates are crystalline. In addition, a key variable side chain is introduced late in the synthesis, allowing analogs to be easily synthesized for optimization of antiviral activity.

## 1. Introduction

Hepatitis C virus (HCV) is an RNA virus which is a very serious human health threat, with an estimated 3% of the world's population being currently infected [1]. No vaccines are available, and the majority of infected people fail to clear the virus and become chronic carriers [2]. In a significant number of people this condition eventually leads to cirrhosis and liver failure. Such HCV-related liver damage is a leading reason for liver transplantations [3].

**Figure 1:**
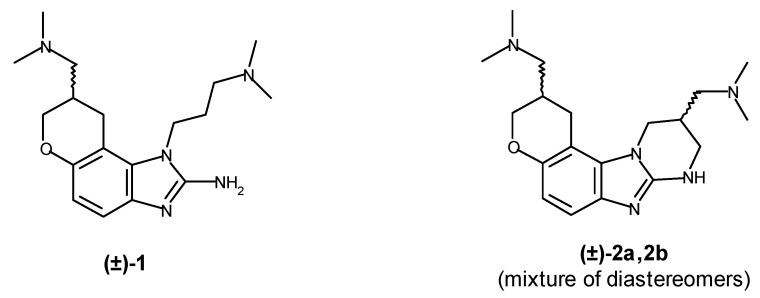
Dihydropyranobenzimidazole inhibitors.

A new and promising HCV drug target is the HCV RNA itself. In 2005, Seth *et al.* [4] reported the discovery of a new class of anti-HCV molecules, benzimidazoles that target the virus by binding to a key part of the 5'-untranslated region of the viral RNA known as IRES (Internal Ribosome Entry Site). Recently, Parsons *et al.* [5] showed that these benzimidazoles inhibit translation initiation through conformational induction. The compounds showed affinity for the HCV RNA as well as inhibitory activity in an HCV replicon assay. The two most potent compounds (Figure 1) possessed an affinity of 0.86 μM and 0.72 μM, respectively, for the key IRES IIa subdomain of the viral RNA [4]. However, the reported synthetic route is lengthy and low-yielding, and the intermediates are troublesome to purify due to the presence of aliphatic amino groups and/or polar side chains. The overall yield of the target compounds is a disappointing 0.6%, making it impractical to synthesize libraries for SAR optimization or to obtain enough material for crystallization studies. Thus, further research into this new mode of anti-HCV activity has been impeded.

In order to circumvent these difficulties and provide access to useful quantities of this class of HCV inhibitors, we have devised a new, efficient synthetic route based on early generation of the chroman nucleus. To avoid difficulties in separating mixtures of diastereomers, we chose to focus our synthetic efforts on a route to (**±**)**-1** rather than (**±**)-**2a,b**. Problematic polar functional groups are introduced in protected form so that all intermediates can be easily purified on silica, and the key *ω*-(dimethylamino)propyl side chain is introduced late in the route, allowing for much easier synthesis of analogous structures for SAR studies.

## 2. Results and Discussion

### 2.1. Chemistry

**Figure 2:**
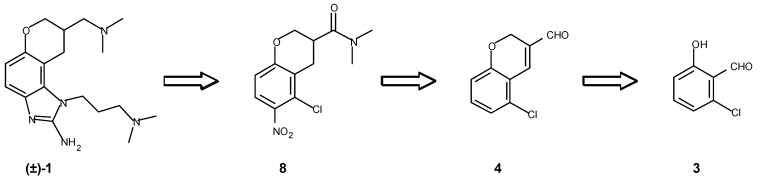
Retrosynthetic analysis.

The first seven steps of the route reported by Seth *et al.* [4] are devoted to stepwise construction of the chroman nucleus. We saw that a considerably more efficient approach would be to create this ring system at the outset. A literature search revealed that Loiodice *et al.* [6] had reported the synthesis of a related chromene in good yield by cyclization of the corresponding salicylaldehyde with acrolein under Baylis-Hillman conditions. Thus a route based on the retrosynthetic analysis given below in Figure 2 appeared feasible.

**Scheme 1:**
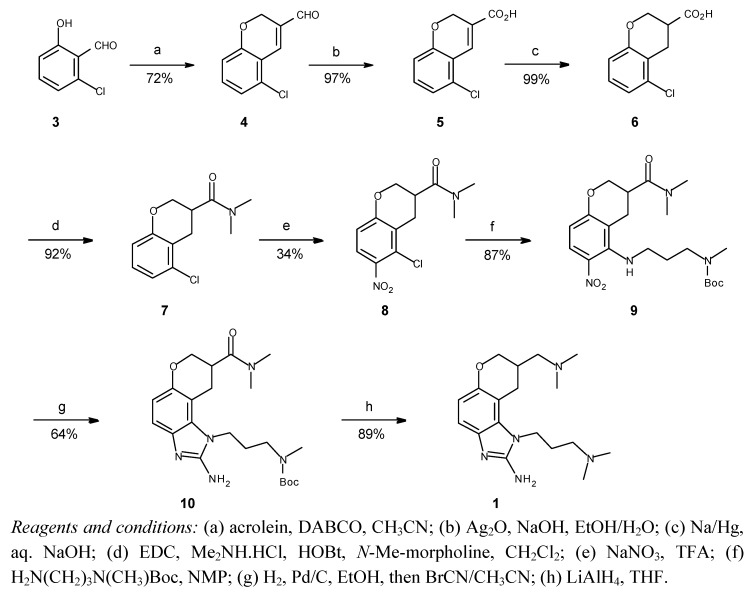
New route to dihydropyranobenzimidazole inhibitors.

Indeed, subjecting readily available 6-chlorosalicylaldehyde **3** [7] to the Baylis-Hillman conditions gave a 72% yield of the desired chromene aldehyde **4** as a crystalline yellow solid. We were thus encouraged to continue development of our envisioned route as shown below in Scheme 1.

The next two steps also take advantage of methods described by Loiodice *et al.* in the previously cited reference [6]. Aldehyde **4** was readily oxidized by freshly generated silver oxide to carboxylic acid **5**, which was then quite cleanly reduced with sodium amalgam under classical conditions to give an excellent yield of chroman **6**. Straightforward EDC-activated coupling of **6** with dimethylamine in the presence of HOBt and *N*-methylmorpholine according to the method of Yoshikawa *et al.* [8] proceeded a bit slowly, but gave an excellent yield of amide **7**. 

**Figure 3:**
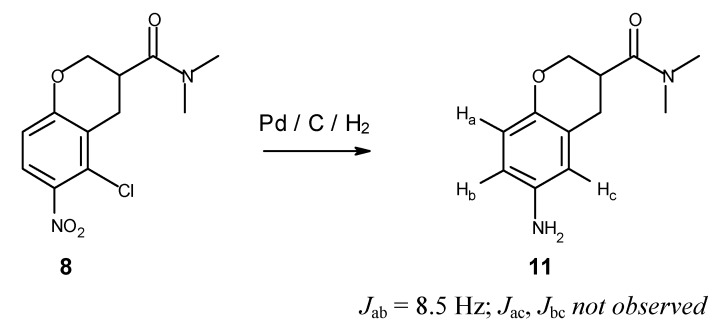
Determination of regiochemistry of nitration based on ^1^H-NMR after hydrogenation.

Nitration with sodium nitrate in trifluoroacetic acid [9] gave a 50% yield of a 2:1 ratio of the desired isomer **8**, in which the nitro group is *para* to the activating alkoxy substituent, and the corresponding *ortho*-substituted minor isomer. Fortunately, the isomers are easily separable by flash chromatography on silica. An attempt to improve the yield by switching to nitronium tetrafluoroborate [10,11] in sulfolane/acetonitrile reversed the regioselectivity, giving mostly the undesired *ortho* isomer. The structure of **8** was verified by palladium-catalyzed reduction/hydrodechlorination to aniline **11** (Figure 3), allowing unambiguous assignment of the position of the nitro substituent based on the observation of only one pair of *ortho*-coupled aryl protons in the NMR spectrum after reduction.

Nucleophilic aromatic substitution of the activated chloro substituent of **8** with commercially available mono-Boc-protected *N*-methyl-1,3-propanediamine [12,13] then gave a nearly quantitative yield of substituted *ortho*-nitroaniline **9**. Adding this side chain at a late point in the synthetic route—only two steps from the end—provides an opportunity for a divergent synthesis of numerous analogs by employing various primary amines for S_N_Ar at this point.

Catalytic hydrogenation of **9** over palladium/charcoal followed immediately by reaction with cyanogen bromide according to the method of Lo *et al.* [14] gave a good yield of aminobenzimidazole **10**, as all other potential nucleophilic sites in the molecule are protected. Then, in the final step of the route, **10** was treated with lithium aluminum hydride in refluxing THF, simultaneously reducing the dimethylamide moiety to an amine and the Boc group of the aminoalkyl side chain to a methyl group to yield the desired product **(±)-1**. The overall yield over eight steps is 10.7%, better than an order of magnitude improvement over the existing published route.

### 2.2. Verification of biochemical activity

The activity of (**±**)**-1** against the HCV IRES domain IIa RNA target was tested in a previously established fluorescence resonance energy transfer (FRET) assay [5] which detects the opening of the bent loop structure of the IIa RNA upon binding of inhibitors. The measured EC_50_ was 2.2 ± 0.4 μM, which roughly corresponds to the value of 0.82 μM reported by Seth *et al.* [4] for the same compound in a mass spectrometry-based assay.

## 3. Experimental

### 3.1. General

Experiments were conducted under an argon atmosphere unless stated otherwise. Reagent grade THF was distilled from sodium/benzophenone. Sodium amalgam was prepared as described by McDonald and Reineke [15]. TLC was performed using EMD Silica Gel 60 F_254_ plates. High-resolution mass spectra were recorded on an Agilent LCTOF high-resolution TOF analyzer with electrospray ionization. Flash chromatography was performed on a Biotage SP1 using prepacked silica columns. Preparative reversed-phase HPLC was performed on a Shimadzu LC-8A HPLC system with a 100 × 30 mm C12 column (Phenomenex Synergi, MAX-RP 80Å, 4 micron), using a water/methanol/0.1% formic acid eluent system with a flow rate of 40 mL/min and detection at 215 nm and 280 nm. ^1^H-NMR and ^13^C-NMR spectra were recorded at 25 °C on a Varian VNMRS-400 spectrometer (^1^H: 400 MHz; ^13^C: 100 MHz), with chemical shift values being reported in ppm relative to tetramethylsilane used as an internal standard. Melting points were measured using a Thomas Hoover Uni-Melt melting point determination apparatus and are uncorrected.

### 3.2. Synthesis

*5-Chloro-2H-chromene-3-carboxaldehyde* (**4**). A mixture of 6-chlorosalicylaldehyde **3** [7] (16.5 g, 0.105 mol), DABCO (5.90 g, 0.053 mol), acrolein (10.5 mL, 0.158 mol), and dioxane (36 mL) was placed in a sealed vial and heated with stirring at 95 °C for 140 minutes. The reaction mixture was cooled to room temperature, diluted with CH_2_Cl_2_, washed with 10% aqueous HCl and then brine, dried with anhydrous Na_2_SO_4_, and evaporated. Chromatography on silica using a gradient of 25–50% CH_2_Cl_2_ in hexanes gave 14.8 g (72%) of **4** as light yellow crystals. An analytical sample was recrystallized from EtOAc/hexanes: m.p. 65.5–66 °C. ^1^H-NMR (DMSO-*d*_6_) δ 9.69 (s, C*H*O, 1H), 7.77 (s, C*H*=CCHO, 1H), 7.34 (dd, Ar*H*, *J* = 8.0, 8.0 Hz, 1H), 7.13 (d, Ar*H*, *J* = 8.0 Hz, 1H), 6.88 (d, Ar*H*, *J* = 8.0 Hz, 1H), 4.94 (s, OC*H*_2_, 2H). ^13^C-NMR (CDCl_3_) δ 189.7, 157.1, 137.0, 133.8, 133.0, 132.3, 122.6, 119.2, 115.3, 63.0. HRMS (M+H)^+^: calc. for C_10_H_8_O_2_Cl, 195.0207; found, 195.0204.

*5-Chloro-2H-chromene-3-carboxylic acid* (**5**). To absolute ethanol (195 mL) in a round-bottomed flask was added a solution of sodium hydroxide (12.2 g, 305 mmol) in water (97 mL). A solution of silver nitrate (27.2 g, 160 mmol) in water (97 mL) was then added dropwise with vigorous stirring. To the resulting suspension of Ag_2_O was added aldehyde **4** (14.8 g), and the mixture was heated and stirred at 85 °C for 75 minutes. The mixture was cooled to room temperature and the clear supernatant was decanted. The solid was washed with a 1:1 ethanol/water solution (3 × 20 mL), and the washings were combined with the decanted supernatant. Dilution with an excess of 1M aq. HCl gave a voluminous white precipitate which dissolved upon extraction with CH_2_Cl_2_. The resulting CH_2_Cl_2_ solution was dried with Na_2_SO_4_, filtered, and evaporated to give 15.5 g (97%) of **5** as cream-colored fluffy crystals. An analytical sample was recrystallized from EtOAc/hexanes: m.p. 192.5–193 °C. ^1^H- NMR (DMSO-*d*_6_) δ 13.10 (bs, CO_2_*H*, 1H), 7.54 (m, C*H*=CCHO, 1H), 7.28 (dd, Ar*H*, *J* = 8.2, 8.2 Hz, 1H), 7.09 (dd, Ar*H*, *J* = 8.2, 1.1 Hz, 1H), 6.87 (ddd, Ar*H*, *J* = 8.2, 1.1, 1.1 Hz, 1H), 4.92 (s, OC*H*_2_, 2H**)**. ^13^C-NMR (CDCl_3_) δ 168.7, 156.6, 133.8, 132.3, 132.1, 122.7, 122.5, 119.4, 115.1, 64.0. HRMS (M–H)^–^: calc. for C_10_H_6_O_3_Cl, 209.0011; found, 209.0012.

*5-Chlorochroman-3-carboxylic acid* (**6**). To a solution of **5** (7.50 g) in 10% aqueous NaOH (193 mL) was added 3% sodium amalgam (103 g) [15]. The mixture was stirred overnight at room temperature. The supernatant was decanted from the liquid mercury, and the mercury was washed twice with small portions of 10% aq. NaOH. The washings were combined with the supernatant, acidified to a pH of 2 with conc. HCl, and extracted with CH_2_Cl_2_. The CH_2_Cl_2_ solution was dried with Na_2_SO_4_, filtered, and evaporated to give 7.51 g (99%) of **6** as a white crystalline solid. An analytical sample was recrystallized from EtOAc/hexanes: m.p. 129.5–130 °C. ^1^H-NMR (DMSO-*d*_6_) δ 12.70 (bs, CO_2_*H*, 1H), 7.10 (dd, Ar*H*, *J* = 8.0, 8.0 Hz, 1H), 6.99 (dd, Ar*H*, *J* = 8.0, 1.2 Hz, 1H), 6.75 (dd, Ar*H*, *J* = 8.0, 1.2 Hz, 1H), 4.28 (dd, OC*H*_2_CH, *J* = 10.8, 4.3 Hz, 1H), 4.16 (dd, OC*H*_2_CH, *J* = 10.8, 7.0 Hz, 1H), 3.05 (m, C*H*CO_2_H, 1H), 2.90 (d, ArC*H*_2_CH, *J* = 6.6 Hz, 2H). ^13^C-NMR (CDCl_3_) δ 178.3, 155.2, 134.6, 127.8, 121.7, 119.0, 115.4, 65.8, 38.2, 25.4. HRMS (M–H)^–^: calc. for C_10_H_8_O_3_Cl, 211.0167; found, 211.0166.

*5-Chloro-N,N-dimethylchroman-3-carboxamide* (**7**). A mixture of **6** (1.50 g, 7.06 mmol), dimethylamine hydrochloride (1.44 g, 17.6 mmol), HOBt (1.43 g, 10.6 mmol), *N*-methylmorpholine (3.88 mL, 35.3 mmol), and EDC hydrochloride (2.03 g, 10.6 mmol) in dichloromethane (65 mL) was stirred at room temperature for 50 hours. The reaction mixture was diluted with additional CH_2_Cl_2_, and an equal volume of saturated aq. NaHCO_3_ was added. The CH_2_Cl_2_ phase was separated, and the aqueous phase was washed 3× with CH_2_Cl_2_. The CH_2_Cl_2_ phases were combined, dried with Na_2_SO_4_, and evaporated. Chromatography on silica (25–75% EtOAc in hexanes) gave 1.56 g (92%) of **7** as a pale yellow oil. ^1^H-NMR (DMSO-*d*_6_) δ 7.12 (dd, Ar-*H*, *J* = 8.2, 8.0 Hz, 1H), 7.01 (dd, Ar-*H*, *J* = 8.0, 1.1 Hz, 1H), 6.79 (dd, Ar-*H*, *J* = 8.2, 1.1 Hz, 1H), 4.31 (m, OC*H*_2_CH, 1H), 3.84 (dd, OC*H*_2_CH, *J* = 10.8, 10.8 Hz, 1H), 3.37-3.30 (m, *partly hidden*, C*H*CONMe_2_, 1H), 3.11 (s, NC*H*_3_, 3H), 2.89 (dd, *partly hidden*, ArC*H*_2_CH, *J* = 16.8, 5.7 Hz, 1H), 2.86 (s, NC*H*_3_, 3H), 2.79 (dd, ArC*H*_2_CH, *J* = 16.8, 10.2 Hz, 1H). ^13^C-NMR (CDCl_3_) δ 171.7, 155.3, 134.6, 127.6, 121.3, 119.9, 115.2, 67.0, 37.2, 35.6, 35.4, 26.9. HRMS (M+H)^+^: calc. for C_12_H_15_NO_2_Cl, 240.0786; found, 240.0792.

*5-Chloro-N,N-dimethyl-6-nitrochroman-3-carboxamide* (**8**). Sodium nitrate (510 mg, 6.00 mmol) was added to a flask containing trifluoroacetic acid (37.5 mL) at 0 °C. After stirring for 10 minutes, the mixture was cooled to –15 °C (by adding small pieces of dry ice to acetone in a Dewar bath) whereupon the solvent began to freeze. With stirring, a solution of **7** (1.25 g, 5.21 mmol) in trifluoroacetic acid (12.5 mL) was added dropwise. An insulating cover was then placed over the Dewar and the mixture was stirred and allowed to warm very slowly to 18 °C over 12 hours. The resulting red solution was quenched with ice, diluted with water, and extracted with dichloromethane. The dichloromethane phase was washed with water, then 5% aq. KH_2_PO_4_ until the pH of the aqueous wash was 4 to 5. The dichloromethane phase was dried with Na_2_SO_4_ and evaporated. The resulting residue was purified on silica (25–75% EtOAc in hexanes) to give 510 mg (34%) of **8** as a yellow oil. ^1^H-NMR (DMSO-*d*_6_) δ 7.86 (d, Ar-*H*, *J* = 9.0 Hz, 1H), 7.00 (d, Ar-*H*, *J* = 9.0 Hz, 1H), 4.39 (m, OC*H*_2_CH, 1H), 4.01 (dd, OC*H*_2_CH, *J* = 11.0, 8.8 Hz, 1H), 3.40 (m, C*H*CO_2_H, 1H), 3.10 (s, NC*H*_3_, 3H), 2.88 (dd, ArC*H*_2_CH, *J* = 17.0, 5.3 Hz, 1H), 2.85 (s, NC*H*_3_, 3H), 2.84 (dd, *partly hidden*, ArC*H*_2_CH, *J* = 17.0, 9.2 Hz, 1H). ^13^C-NMR (CDCl_3_) δ 170.8, 158.2, 141.6, 128.7, 124.9, 122.1, 115.4, 67.4, 37.3, 35.7, 34.7, 27.3. The undesired minor *ortho* isomer, which eluted just before compound **8**, was also isolated: 250 mg (17%) of *5-chloro-N,N-dimethyl-8-nitrochroman-3-carboxamide* as a pale yellow oil, ^1^H-NMR (DMSO-*d*_6_) δ 7.79 (d, Ar-*H*, *J* = 8.8 Hz, 1H), 7.21 (d, Ar-*H*, *J* = 8.8 Hz, 1H), 4.48 (m, OC*H*_2_CH, 1H), 4.06 (dd, OC*H*_2_CH, *J* = 10.9, 9.1 Hz, 1H), 3.46 (m, C*H*CO_2_H, 1H), 3.11 (s, NC*H*_3_, 3H), 2.95 (dd, ArC*H*_2_CH, *J* = 17.0, 5.8 Hz, 1H), 2.86 (s, NC*H*_3_, 3H), 2.86 (dd, *partly hidden*, ArC*H*_2_CH, *J* = 16.7, 10.0 Hz, 1H). ^13^C-NMR (CDCl_3_) δ 170.5, 149.3, 139.8, 137.6, 124.0, 123.1, 120.6, 67.8, 37.2, 35.7, 34.4, 27.2.

*tert-Butyl N-[3-[[3-(dimethylcarbamoyl)-6-nitrochroman-5-yl]amino]propyl]-N-methylcarbamate* (**9**). A mixture of **8** (300 mg, 1.05 mmol), *tert*-butyl *N*-(3-aminopropyl)-*N*-methylcarbamate (989 mg, 5.25 mmol), and *N*-methylpyrrolidinone (989 mg) was heated in a sealed tube at 75 °C for 22 hours. The reaction mixture was diluted with Et_2_O (75 mL) and washed with H_2_O (4 × 75 mL) and 5% aq. KH_2_PO_4_ (75 mL), then dried with Na_2_SO_4_, filtered, and evaporated. The crude product was purified on silica using a gradient of 40–50% EtOAc in CH_2_Cl_2_ to give 399 mg (87%) of **9** as a bright yellow-orange oil. ^1^H-NMR (DMSO-*d*_6_) δ 7.82 (d, Ar-*H*, *J* = 9.4 Hz, 1H), 7.20 (b, N*H*, 1H), 6.35 (d, Ar-*H*, *J* = 9.4 Hz, 1H), 4.37 (m, OC*H*_2_CH, 1H), 3.93 (dd, OC*H*_2_CH,*J* = 10.6, 10.6 Hz, 1H), 3.25 (m, *partly hidden*, C*H*CO_2_H, 1H), 3.25–3.10 (m, 2 × NC*H*_2_, 4H), 3.06 (s, NC*H*_3_, 3H), 2.85 (s, NC*H*_3_, 3H), 2.80–2.70 (m, *partly hidden*, ArC*H*_2_CH, 2H), 2.71 (s, NC*H*_3_, 3H), 1.70 (qu, CH_2_C*H*_2_CH_2_, *J* = 6.8 Hz, 2H), 1.34 (s, *t*-Bu, 9H). ^13^C-NMR (CDCl_3_) δ 171.4, 160.1, 155.7, 148.8, 131.6, 126.5, 111.0, 109.3, 79.6, 77.2, 67.4, 45.8, 37.3, 35.7, 35.3, 34.4, 29.4, 28.5, 27.9. HRMS (M+H)^+^: calc. for C_21_H_33_N_4_O_6_, 437.2395; found, 437.2401.

*tert-Butyl N-[3-[2-amino-8-(dimethylcarbamoyl)-8,9-dihydro-7H-pyrano[2,3-g]benzimidazol-1-yl]- propyl]-N-methylcarbamate* (**10**). To a flask containing 10% palladium on carbon (180 mg) was added a solution of **9** (187 mg, 0.43 mmol) in absolute ethanol (20 mL). The mixture was stirred for 90 minutes under 1 atmosphere of hydrogen at room temperature, at which point ^1^H-NMR analysis showed complete reduction to the corresponding aniline derivative. The mixture was filtered through Celite. The Celite was washed with three 5-mL aliquots of acetonitrile and all filtrates were combined. To this solution was added cyanogen bromide (49 mg) in acetonitrile (2 mL), and the mixture was stirred at room temperature for 15 hours under argon. The solvent was evaporated, and the residue was dissolved in DMSO (1 mL) and purified by preparative HPLC using a gradient of 5–50% methanol/water/0.1% formic acid over 15 minutes, followed by a ramp to 100% methanol/0.1% formic acid over 3 minutes. The fractions containing the major peak were combined and neutralized with aq. NaHCO_3_. Most of the methanol was evaporated *in vacuo*, and the resulting aqueous phase was extracted with CH_2_Cl_2_. Drying with Na_2_SO_4_, filtration, and evaporation gave 119 mg (64%) of **10** as a nearly colorless oil which solidified into a light tan crystalline solid. An analytical sample was recrystallized from EtOAc/hexanes: m.p. 165.5–166 °C. ^1^H-NMR (DMSO-*d*_6_) δ 6.86 (d, Ar-*H*, *J* = 8.4 Hz, 1H), 6.43 (d, Ar-*H*, *J* = 8.4 Hz, 1H), 6.09 (bs, Ar-N*H*_2_, 2H), 4.23 (dd, OC*H*_2_CH, *J* = 10.6, 2.93 Hz, 1H), 4.03 (bm, NC*H*_2_CH_2_, 2H), 3.78 (dd, OC*H*_2_CH, *J* = 10.4, 10.4 Hz, 1H), 3.35–3.10 (m, *major overlap*, C*H*CONMe_2_, ArC*H*_2_CH, C*H*_2_NMe, 5H), 3.12 (s, NC*H*_3_, 3H), 2.88 (s, NC*H*_3_, 3H), 2.76 (s, NC*H*_3_, 3H), 1.81 (m, CH_2_C*H*_2_CH_2_, 2H), 1.33 (bs, *t*-Bu, 9H). ^13^C-NMR (DMSO-*d*_6_) δ 171.9, 154.9, 148.1, 137.1, 132.0, 114.1, 109.9, 104.5, 78.9, 66.6, 46.2, 45.7, 41.6, 37.2, 35.5, 35.2, 34.1, 29.3, 28.4, 24.3. HRMS (M+H)^+^: calc. for C_22_H_34_N_5_O_4_, 432.2605; found, 432.2621.

*6-Amino-N,N-dimethylchroman-3-carboxamide* (**11**). Compound **8** (42 mg) was dissolved in absolute ethanol (10 mL) and stirred with 10% palladium on carbon (65 mg) under 1 atm of hydrogen at 50 °C for 3 hours. The mixture was filtered through Celite, and the absorbent was washed several times with ethanol. The combined filtrates were evaporated to give a quantitative yield of **11** as a tan solid. ^1^H- NMR (DMSO-*d*_6_) δ 8.71 (bs, Ar-N*H*_2_, 2H), 6.86 (s, Ar-*H*, 1H), 6.84 (d, Ar-*H*, *J* = 8.5 Hz, 1H), 6.73 (d, Ar-*H*, *J* = 8.5 Hz, 1H), 4.26 (m, OC*H*_2_CH, 1H), 3.81 (dd, OC*H*_2_CH, *J* = 10.9, 10.0 Hz, 1H), 3.23 (m, C*H*CO_2_H, 1H), 3.08 (s, NC*H*_3_, 3H), 2.86 (dd, *partly hidden*, ArC*H*_2_CH, *J* = 16.1, 10.6 Hz, 1H), 2.84 (s, NC*H*_3_, 3H), 2.79 (dd, ArC*H*_2_CH, *J* = 16.4, 5.4 Hz, 1H).

*8-(Dimethylaminomethyl)-1-(3-dimethylaminopropyl)-8,9-dihydro-7H-pyrano[3,2-e]benzimidazol-2-amine* (**1**). Compound **10** (100 mg, 0.232 mmol) was dissolved in THF (5 mL) under argon. Lithium aluminum hydride (333 mg, 8.77 mmol) was added and the mixture was stirred at 60 °C for 1.5 hours, then quenched by careful dropwise addition of 3M aq. NaOH. The solids were filtered off and washed with THF. The filtrate and washings were combined, dried with Na_2_SO_4_, and evaporated to give a tan solid, which was triturated with a minimal amount of EtOAc to give 68 mg (89%) of **1** as an off-white crystalline solid: m.p. 179.5–180.5 °C. ^1^H-NMR (DMSO-*d*_6_) δ 6.83 (d, Ar-*H*, *J* = 8.4 Hz, 1H), 6.39 (d, Ar-*H*, *J* = 8.4 Hz, 1H), 6.14 (bs, Ar-N*H*_2_, 2H), 4.13 (d, OC*H*_2_CH, *J* = 9.4 Hz, 1H), 4.04 (dd, NC*H*_2_CH_2_, *J* = 6.9, 6.9 Hz, 2H), 3.72 (dd, OC*H*_2_CH, *J* = 10.2, 7.2 Hz, 1H), 3.16 (dd, ArC*H*_2_CH, *J* = 16.0, 4.5 Hz, 1H), 2.76 (dd, ArC*H*_2_CH, *J* = 16.0, 7.2 Hz, 1H), 2.30-2.20 (m, *major overlap*, C*H*CONMe_2_, 2 × C*H*_2_NMe_2_, 5H), 2.17 (s, NMe_2_, 6H), 2.16 (s, NMe_2_, 6H), 1.80 (m, CH_2_C*H*_2_CH_2_, 2H). ^13^C-NMR (CDCl_3_) δ 155.2, 149.1, 136.7, 132.2, 115.1, 110.9, 104.0, 68.5, 61.8, 53.8, 46.0, 44.4, 39.9, 30.6, 29.0, 25.6. HRMS (M+H)^+^: calc. for C_18_H_30_N_5_O, 332.2445; found, 332.2453.

### 3.3. FRET assay

The FRET assay was conducted using a cyanine dye-labeled HCV IRES domain IIa RNA construct as previously described [5]. Briefly, the RNA construct was titrated with increasing amounts of compound **1** and the dose response of the FRET efficiency was recorded. Titrations were performed in triplicate. Fitting of the average FRET signal at each concentration of compound to a one-site binding model resulted in an EC_50_ of 2.2 ± 0.4 μM for compound **1** binding to the IIa target.

## 4. Conclusions

We have achieved an efficient new synthesis of the potent HCV inhibitor (**±**)**-1** in eight steps and 10.7% overall yield from readily available 6-chlorosalicylaldehyde. This represents better than a fifteen-fold improvement over the published route. In addition, most intermediates are crystalline and easily purified. The high efficiency of the new route combined with the late-stage introduction of the aminoalkyl side chain enables the practical and rapid generation of analogs for SAR studies. We are currently generating new analogs using this route and will report our findings in a subsequent publication.
